# High-sensitivity cardiac troponin T is more helpful in detecting peri-operative myocardial injury and apoptosis during coronary artery bypass graft surgery

**DOI:** 10.5830/CVJA-2015-052

**Published:** 2015

**Authors:** Emel Fatma Kocak, Irfan Altuntas, Cengiz Kocak, Ahmet Aksoy, Ibrahim Fevzi Ozdomanic, Ozden Ozben Isiklar, Raziye Akcilar, Cevher Unsal, Merve Celenk

**Affiliations:** Department of Medical Biochemistry, Faculty of Medicine, Dumlupinar University, Kutahya, Turkey; Department of Medical Biochemistry, Faculty of Medicine, Dumlupinar University, Kutahya, Turkey; Department of Pathology, Faculty of Medicine, Dumlupinar University, Kutahya, Turkey; Department of Cardiovascular Surgery, Evliya Celebi Education and Research Hospital, Dumlupinar University, Kutahya, Turkey; Department of Cardiovascular Surgery, Evliya Celebi Education and Research Hospital, Dumlupinar University, Kutahya, Turkey; Department of Medical Biochemistry, Evliya Celebi Education and Research Hospital, Dumlupinar University, Kutahya, Turkey; Department of Physiology, Faculty of Medicine, Dumlupinar University, Kutahya, Turkey; Department of Anesthesiology and Reanimation, Evliya Celebi Education and Research Hospital, Dumlupinar University, Kutahya, Turkey; Department of Medical Biochemistry, Evliya Celebi Education and Research Hospital, Dumlupinar University, Kutahya, Turkey

**Keywords:** apoptosis, creatine kinase, MB form, coronary artery bypass, myocardial reperfusion injury, troponin

## Abstract

**Aim:**

To determine whether there is a correlation between cardiac markers and peri-operative myocardial injury (PMI) and apoptosis in coronary artery bypass graft (CABG) surgery and to compare the efficacy of cardiac markers to detect PMI.

**Methods:**

The study population consisted of 37 patients (24 male, 13 female, mean age 63.4 ± 8.9 years) undergoing elective CABG. Arterial and coronary sinus blood samples were collected just before aortic cross-clamping (pre-ACC) and after aortic declamping (post-ACC). Creatine kinase-MB isoenzyme (CK-MB) activity, and high-sensitivity cardiac troponin T (hs-cTnT), creatine kinase-MB isoenzyme mass (CK-MB mass) and cardiac troponin I (cTnI) concentrations were measured in blood samples. Myocardial injury and apoptosis were examined in atrial biopsies.

**Results:**

CABG caused PMI and apoptosis in all cases. Concentrations and net releases of cardiac markers significantly increased after aortic declamping (*p* < 0.001 for CK-MB and CK-MB mass, *p* < 0.01 for cTnI, *p* < 0.05 for hs-cTnT). A positive correlation was found between apoptotic index (*r* = 0.611, *p* < 0.001 for cTnI; r = 0.806, *p* < 0.001 for hs-cTnT), myocardial injury score (*r* = 0.544, *p* < 0.001 for cTnI; *r* = 0.719, *p* < 0.001 for hs-cTnT) and cTnI and hs-cTnT values in the post-ACC period. A positive correlation was found between apoptotic index (*r* = 0.507, *p* < 0.001), myocardial injury score (*r* = 0.416, *p* = 0.010) and net release of hs-cTnT. Furthermore, a positive correlation was found between aortic cross-clamp (ACC) time (*r* = 0.448, *p* = 0.007), cardiopulmonary bypass (CPB) time (*r* = 0.342, *p* = 0.047) and net release of hs-cTnT.

**Conclusion:**

Although both cTnI and hs-cTnT may be specific and efficacious markers of myocardial apoptosis and injury occurring during CABG with CPB, hs-cTnT may be a more useful marker than cTnI to detect peri-operative myocardial apoptosis and injury.

## Aim

Coronary artery bypass grafting (CABG) accompanied by cardiopulmonary bypass (CPB) is a safe, routine procedure for the surgical treatment of various heart diseases, including coronary artery disease. Cardiopulmonary bypass and cardioplegic arrest enable the performance of coronary artery anastomosis in a bloodless and motionless field during CABG surgery.^1^,^2^ However, peri-operative myocardial injury (PMI) is a major problem and the most common cause of morbidity and mortality during CABG surgery.^3^

Despite optimal myocardial protective techniques, a certain amount of myocardial injury may occur in the majority of patients undergoing CABG surgery. Various factors can cause myocardial injury during CABG surgery, most importantly CPB, surgical technique, suture placement or manipulation of the heart, coronary dissection, aortic cross-clamping (ACC), and 2inadequate cardiac protection.^4^-^6^

During CPB, the heart is arrested and protected by cardioplegia. This period is associated with oxygen deprivation and the heart is ischaemic during this time. At the end of CPB, the heart is reperfused and cardiac action resumes. These ischaemic and subsequent reperfusion periods cause myocardial injury and even necrosis.^7^ Myocardial ischaemia causes intracellular calcium accumulation and degradation of the membrane lipids, and oedema during ACC. After removal of the aortic cross-clamp, reperfusion causes oxidative stress depending on the production of reactive oxygen (ROS) and reactive nitrogen species (RNS).^8^ In addition, it has been reported that myocardial ischaemia–reperfusion (I/R) induces cardiomyocytic apoptosis.^9^,^10^

Early and accurate detection of PMI may prompt immediate improvement in the perfusion and oxygen demand of the myocardium,which may limit PMI. Therefore, it is important to have a highly specific diagnostic marker to detect PMI.

Cardiac surgery may lead to the release of markers of myocardial injury. Interpretation of these elevated cardiac markers in the blood during the peri-operative period is confusing because increases in cardiac markers may be related to direct skeletal muscle injury due to the surgical procedure, or to myocardial I/R injury. It is diffucult to differantiate between increases in cardiac markers related to surgical procedure and pathological myocardial I/R injury.^11^

In this study, we considered that histopathological examination of myocardial tissue would clearly reveal myocyte damage occurring in the peri-operative period, and increases in cardiac markers could be properly interpreted, comparing them with the results of the histopathological examination. To the best of our knowledge, the relationship between severity of PMI and apoptosis, and the cardiac markers assayed in this study has not been previously studied in CABG surgery with CPB.

This study therefore had the following objectives: (1) to examine whether PMI, as occurs during CABG surgery, is associated with myocardial apoptosis and the release of cardiac markers, using biochemical and histopathological analysis; (2) to determine whether there is a direct relationship between the release of cardiac markers and the severity of myocardial injury and apoptosis, as graded histopathologically; and (3) to compare efficacies of cardiac markers to detect PMI rapidly and accurately

## Methods

This prospective study was carried out in Dumlupinar University Evliya Celebi Research and Education Hospital, Turkey, between April and September 2014. The study was in accordance with the principles outlined in the Declaration of Helsinki. Ethical approval was received from the local Human Research Ethics Committee (no: 2013/14-122). Written informed consent was obtained from the all patients.

The study population consisted of 37 patients (24 male, 13 female, mean age 63.4 ± 8.9 years) undergoing elective CABG who fulfilled the inclusion criteria. Inclusion criteria were age over 18 and less than 80 years, and need for elective myocardial revascularisation for angina pectoris. The exclusion criteria included ejection fraction < 30%; recent anterior myocardial infarction (< one month), the requirement of a concomitant cardiac operation, emergency surgery or re-operation. Demographic, pre-operative and intra-operative data of patients are shown in Table 1.

**Table 1:** Demographic, pre-operative and intra-operative data of the patients

*Parameters*	*n = 37*
Age (years)	63.4 ± 8.9
Male (n)	24
Female (n)	13
Weight (kg)	75.8 ± 13.7
Height (cm)	162.7 ± 8.6
BMI (kg/m2)	28.67 ± 4.8
NYHA classification (n)	
Class I	21
Class II	14
Class III	2
LVEF (%)	
Normal (> 50%)	23
Moderate (31–49%)	14
MI history (n)	15
Medication (n)	
β-Blockers	33
ACE inhibitors	8
Calcium antagonists	11
Statins	30
Acetylsalicylic acid	35
Other anticoagulants	4
ACC time (min)	56.7 ± 15.3
CPB time (min)	101.9 ± 23.4
Grafted vessels (n)	3.17 ± 0.62
Apoptotic index (TUNEL, %)	25.7 ± 8.4
Myocardial injury score	1.5 ± 0.5

BMI: body mass index; NYHA: New York Heart Association; LVEF: left ventricular ejection fraction; MI: myocardial infarction; ACE: angiotensin converting enzyme; ACC: aortic cross-clamping; CPB: cardiopulmonary bypass; TUNEL: terminal deoxynucleotidyl transferase-mediated deoxyuridine triphosphate nick end-labelling; SD: standard deviation. Data are presented as median ± SD or number.

## Anesthesia, CPB and surgical procedure

The same surgical and anesthetic team managed all patients. Cardiopulmonary bypass and surgical techniques were standardised and did not change during the study period. Pre-medication, general anaesthesia with endotracheal intubation, and transfusions were the same in all cases. Induction of anaesthesia was performed using 5–10 mcg/kg fentanyl, 3–5 mcg/kg thiopental, 0.05 mg/kg midazolam and 0.1 mg/kg vecuronium. Anaesthesia was maintained using 2% sevoflurane and 1–3 mcg/kg/dk remifentanil.

A median sternotomy was performed with a midsternal incision, followed by routine aortic and right atrial cannulation. After harvesting the bypass graft conduits (left internal mammary artery and saphenous vein) the patients were prepared for CPB. Anticoagulation was achieved with 400 U/kg heparin. CPB was carried out using membrane oxygenators and moderate systemic hypothermia.

Myocardial protection was achieved with combined antegrade and retrograde continuous mild hypothermic (32°C) blood cardioplegia. The contents of the cardioplegia solution were as follows: 80 mEq potassium, 12 mEq magnesium and 44 mEq sodium bicarbonate in 0.9% saline, and this solution was diluted with blood in a ratio of 1:4.

Aortic cross-clamping was performed and diastolic arrest was achieved by cardioplegia. After the distal anastomoses were completed, the aortic cross-clamp was removed and the proximal anastomoses were performed on the aorta during myocardial perfusion. Before releasing the aortic cross-clamp, warm reperfusion was given (37°C) until the patient’s body temperature was 35–37°C. Heparin was neutralised with protamine in a ratio of 1:1.5 within 10 minutes of the end of CPB.

## Blood sample collection and measurement of cardiac markers

Blood samples were drawn after atrial cannulation, just before aortic cross-clamping (pre-ischaemic sample), and within 15 minutes of aortic declamping (reperfusion sample). Blood samples were collected from the arterial line of the bypass circuit (arterial sample) and from the pressure-monitoring line of the coronary sinus perfusion catheter (coronary sinus sample).

Blood samples were collected into an evacuated serumseparator clot-activator tube (Vacuette®, Greiner Bio-One, Kremsmunster, Austria) and a 2.0-ml dipotassium (K_2_) ethylene diamine tetra-acetic acid (EDTA) vacuum tube (BD Vacuteiner® BD Plymouth, UK) for creatine kinase-MB isoenzyme (CK-MB), high-sensitivity cardiac troponin T (hs-cTnT), creatine kinase-MB isoenzyme mass (CK-MB mass) and cardiac troponin I (cTnI) measurements. The tubes were centrifuged at 1 500 × g for 15 minutes within one hour to obtain serum samples for the measurement of CK-MB and hs-cTnT concentrations.

Whole blood samples, which were collected in the K_2_ EDTA tubes, were not centrifuged and CK-MB mass and cTnI concentrations were measured in the whole blood samples on the same day as the surgery.

Serum CK-MB activities were measured with the immunoinhibition method on a Roche Cobas c501 analyser (Roche Diagnostics GmbH, Mannheim, Germany). The reference range of CK-MB activity measured by this method was < 25 U/l.

Serum hs-cTnT concentrations were measured by electrochemiluminescence immunoassays (ECLIA) on a Roche Cobas e 601 analyser (Roche Diagnostics GmbH, Mannheim, Germany). In healthy subjects, the upper reference limit for hs-cTnT concentrations was 14 ng/l (99th percentile) and the measurement range was 3–10 000 ng/l.

CK-MB mass and cTnI were measured with the time-resolved fluorescence method on a radiometer AQT90 FLEX (Radiometer Medical ApS, Brønshøj, Denmark). In healthy subjects the upper reference limit for cTnI concentrations was 0.023 µg/l (99th percentile) and the measurement range was 0.010–25 µg/l. In healthy subjects the upper reference limit for CK-MB mass concentrations was < 7.2 µg/l (99th percentile) and the measurement range was 2–500 µg/l.

## Atrial tissue sample collection and histopathological examinations

Using a sharp scalpel, myocardial biopsy samples from the same site of the right atrial appendage were taken from each patient within 15 minutes of aortic declamping. The area of the biopsy sample in contact with the forceps was removed. Particular care was taken to avoid possible ischaemic areas caused by surgical manipulation. Tissue samples were fixed in 10% formalin, embedded in paraffin, sectioned (4 µm), placed on slides, stained with haematoxylin and eosin (H&E), and examined under a light microscope (Olympus BX51, Tokyo, Japan) by a pathologist who was blinded to the study design.

The slides were graded histopathologically, according to the severity of myocardial injury, using a previously described scoring system.^12^ Histological changes (oedema, leukostasis, cell necrosis and focal bleeding) were scored from 0 to 3 as follows: 0 = no changes; 1 = slight changes: focal myocyte damage or small multifocal degeneration with slight degree of inflammation; 2 = moderate changes: extensive myofibrillar degeneration and/or diffuse inflammatory process; 3 = severe changess: necrosis with diffuse inflammatory process.

## In situ detection of myocardial apoptosis

We used an *in situ* TUNEL (terminal deoxynucleotidyl transferase-mediated deoxyuridine triphosphate nick end-labelling) assay to assess the degree of myocardial apoptosis. Formalin-fixed sections were deparaffinised in xylene and rehydrated through graded concentrations of ethanol to water.

DNA fragmentation during apoptosis was detected using a commercially available kit (ApopTag® peroxidase *in situ* apoptosis detection kit, Millipore, Billerica, MA, USA) according to the manufacturer’s instructions. Processed samples were examined under a light microscope (Olympus BX51, Tokyo, Japan). For quantitative analysis, TUNEL-positive cells were counted in six random fields per section (80–120 cells per field). The apoptotic index was calculated as the mean of apoptotic (positive-stained) cells.

## Statistical analyses

Statistical analyses were performed using GraphPad Prism version 6.05 (GraphPad Software, Inc, CA, USA). All data sets were tested for normality using the Shapiro–Wilk test. Data were presented as median and interquartile ranges (IQR) and non-parametric statistical tests were used, as the values were not normally distributed. The net release of cardiac markers was quantified as the arteriovenous difference (coronary sinus concentration minus arterial concentration).

The comparison of cardiac marker values between pre-ACC (just before aortic cross-clamping) and post-ACC (within 15 minutes of aortic declamping) periods was analysed using the Mann–Whitney *U*-test. The correlation between apoptotic index (TUNEL), histopathological myocardial injury score, intra-operative data and cardiac marker values in the post-ACC period was analysed using Spearman’s correlation analysis. An r-value > 0.5 indicated a strong correlation, 0.35–0.5 a moderate correlation, and 0.2–0.34 a weak correlation.^13^ A *p*-value < 0.05 was considered statistically significant.

## Results

**Fig. 1.**
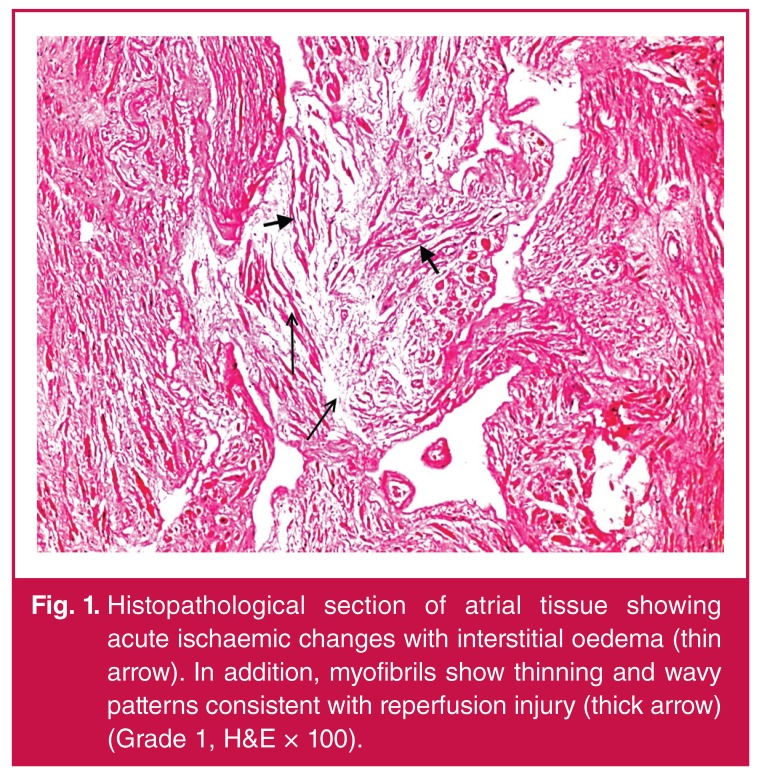
Histopathological section of atrial tissue showing acute ischaemic changes with interstitial oedema (thin arrow). In addition, myofibrils show thinning and wavy patterns consistent with reperfusion injury (thick arrow) (Grade 1, H&E × 100).

**Fig. 2.**
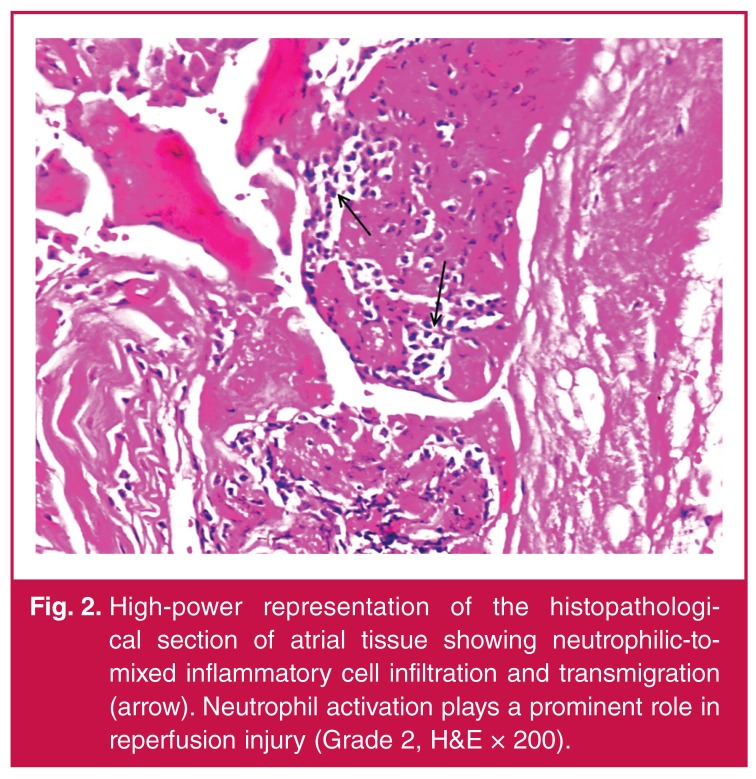
High-power representation of the histopathological section of atrial tissue showing neutrophilic-tomixed inflammatory cell infiltration and transmigration (arrow). Neutrophil activation plays a prominent role in reperfusion injury (Grade 2, H&E × 200).

**Fig. 3.**
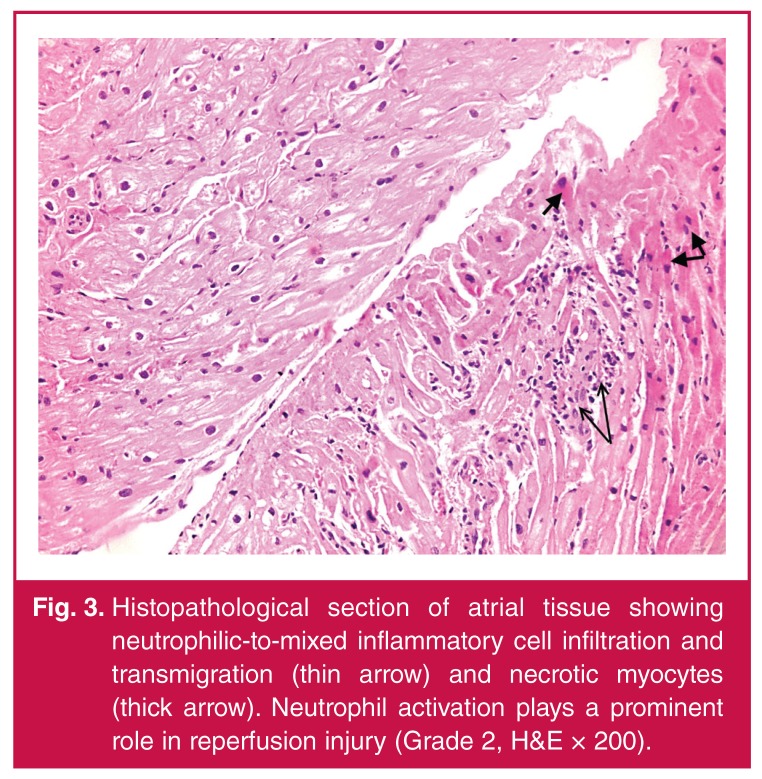
Histopathological section of atrial tissue showing neutrophilic-to-mixed inflammatory cell infiltration and transmigration (thin arrow) and necrotic myocytes (thick arrow). Neutrophil activation plays a prominent role in reperfusion injury (Grade 2, H&E × 200).

**Fig. 4.**
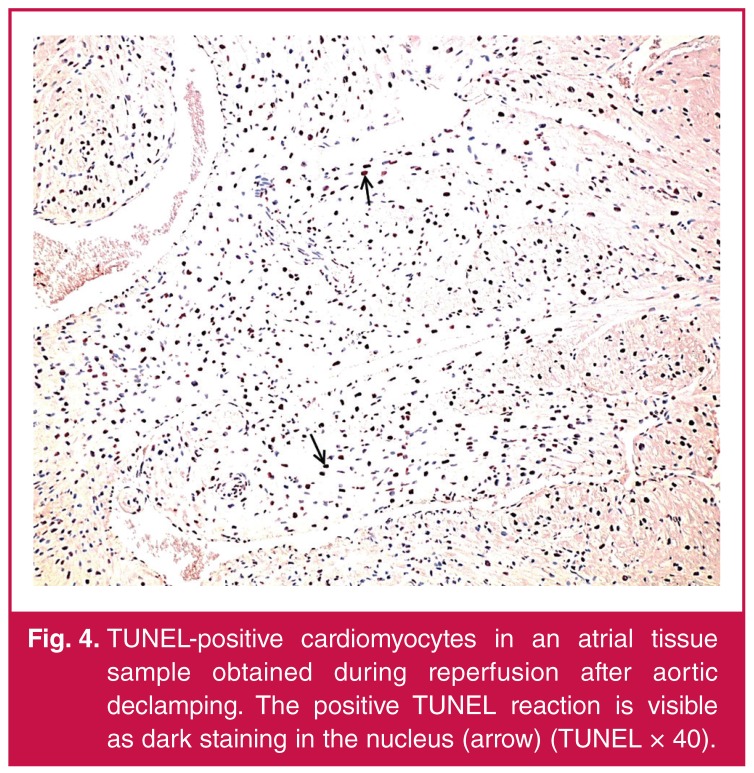
TUNEL-positive cardiomyocytes in an atrial tissue sample obtained during reperfusion after aortic declamping. The positive TUNEL reaction is visible as dark staining in the nucleus (arrow) (TUNEL × 40)..

Demographic, pre-operative and intra-operative data of the patients are shown in Table 1. In the histopathological examinations, our results showed that CABG surgery with CPB and ACC caused slight-to-moderate myocardial injury and moderate-to-severe apoptosis in all cases (Table 1). Acute ischaemic changes with interstitial oedema, myofibrillar thinning and wavy pattern consistent with reperfusion injury were observed in histopathological sections of atrial tissue. In addition, neutrophilic-to-mixed inflammatory cell infiltration and transmigration indicating reperfusion injury were observed (Fig. 1, Fig. 2, Fig. 3, Fig. 4).

When the cardiac marker values in arterial and coronary sinus blood samples were compared, significant differences were found between the pre-ACC and post-ACC periods (Table 2). On the other hand, when net release of cardiac markers was compared, significant differences were found between the pre-ACC and post-ACC periods for CK-MB, CK-MB mass, cTnI and hs-cTnT (Table 3).

**Table 2:** Comparison of the cardiac marker values in arterial and coronary sinus blood samples between the pre-ACC and post-ACC period

*Cardiac markers*	*Pre-ACC arterial blood*	*Post-ACC arterial blood*	*p-value*	*Pre-ACC coronary sinus blood*	*Post-ACC coronary sinus blood*	*p-value*
CK-MB (U/l)	16.4	42.0	< 0.001	19.7	51.5	< 0.001
	(12.0–21.0)	(32.0–73.0)		(14.8–24.0)	(35.7–85.5)	
CK-MB mass (µg/l)	6.5	21.0	< 0.001	7.3	25.0	< 0.001
	(5.5–10.4)	(15.5–28.0)		(6.0–12.5)	(17.5–34.0)	
cTnI (µg/l)	0.12	0.25	< 0.01	0.14	0.31	< 0.01
	(0.07–0.3)	(0.13–0.42)		(0.08–0.29)	(0.17–0.49)	
hs-cTnT (ng/l)	125.0	193.0	< 0.05	159.0	239.0	< 0.05
	(59.5–211.6)	(91.0–309.0)		(66.0–230.7)	(95.5–425.0)	

ACC: aortic cross-clamping; CK-MB: creatine kinase isoenzyme MB; cTnI: cardiac troponin I; hs-cTnT: high-sensitivity cardiac troponin T. Data are presented as median and interquartile ranges for each group. Data were tested using the Mann– Whitney *U*-test. A *p*-value < 0.05 was considered statistically significant.

**Table 3:** Comparison of the net release of cardiac markers between the pre-ACC and post-ACC period

*Cardiac markers*	*Pre-ACC net release*	*Post-ACC net release*	*p-value*
CK-MB (U/l)	3.0	7.0	< 0.001
(1.8–5.0)	(2.9–15.0)		
CK-MB mass (µg/l)	1.0	2.0	< 0.001
	(0.5–1.4)	(2.0–4.0)	
cTnI (µg/l)	0.02	0.03	< 0.01
	(0.01–0.04)	(0.02–0.06)	
hs-cTnT (ng/l)	15.0	26.0	< 0.05
	(5.4–42.0)	(9.5–79.6)	

The relationship between apoptotic index (TUNEL), histopathological myocardial injury score and cardiac marker values in arterial and coronary sinus blood ACC: aortic cross-clamping; CK-MB: creatine kinase isoenzyme MB; cTnI: cardiac troponin I; hs-cTnT: high-sensitivity cardiac troponin T; pre-ACC: just before aortic cross-clamping; post-ACC: within 15 minutes of aortic declamping. The net releases of cardiac markers were quantified as the arteriovenous difference (coronary sinus concentration minus arterial concentration). Data are presented as median and interquartile ranges for each group. Data were tested using the Mann–Whitney *U*-test. A *p*-value < 0.05 was considered statistically significant.samples in the post-ACC period

A significant positive correlation was observed between apoptotic index and arterial blood CK-MB mass, cTnI and hs-cTnT values in the post-ACC period. A positive correlation was observed between the apoptotic index and coronary sinus blood CK-MB mass, cTnI and hs-cTnT values in the post-ACC period. No correlation was found between apoptotic index and arterial and coronary sinus blood CK-MB values (Table 4).

**Table 4:** The relationship between apoptotic index (TUNEL), histopathological myocardial injury score and cardiac marker values in arterial and coronary sinus blood samples in the post-ACC period

**	*CK-MB (U/l)*	*CK-MB mass (µg/l)*	*cTnI (µg/l)*	*hs-cTnT(ng/l)*
Arterial blood samples				
Apoptotic index	r = 0.019	r = 0.422	r = 0.611	r = 0.809
(TUNEL)	p = 0.910	p = 0.009*	p < 0.001*	p < 0.001*
Myocardial	r = 0.021	r = 0.316	r = 0.544	r = 0.719
injury score	p = 0.900	p = 0.057	p < 0.001*	p < 0.001*
Coronary sinus blood samples				
Apoptotic index	r = 0.085	r = 0.358	r = 0.623	r = 0.790
(TUNEL)	p = 0.616	p = 0.030*	p < 0.001*	p < 0.001*
Myocardial	r = 0.087	r = 0.223	r = 0.554	r = 0.695
injury score	p = 0.606	p = 0.184	p < 0.001*	p < 0.001*

ACC: aortic cross-clamping; CK-MB: creatine kinase isoenzyme MB; cTnI: cardiac troponin I; hs-cTnT: high-sensitivity cardiac troponin T; TUNEL: terminal deoxynucleotidyl transferase-mediated deoxyuridine triphosphate nick end-labelling; post-ACC: within 15 minutes of aortic declamping. Relationships between data were tested using Spearman’s correlation analysis. *A p-value < 0.05 was considered statistically significant.

A significant positive correlation was observed between myocardial injury score and arterial blood cTnI and hs-cTnT values in the post-ACC period. In addition, a positive correlation was found between myocardial injury score and coronary sinus blood cTnI and hs-cTnT values in the post-ACC period. No correlation was observed between myocardial injury score and arterial and coronary sinus blood CK-MB and CK-MB mass values (Table 4).

When the relationship between apoptotic index and net release of cardiac markers in the post-ACC period was analysed, a positive correlation was found between apoptotic index and net release of hs-cTnT. No correlation was found between apoptotic index and net release of CK-MB, CK-MB mass and and cTnI (Table 5). A positive correlation was found between myocardial injury score and net release of cTnI and hs-cTnT in the post-ACC period. No correlation was found between myocardial injury score and net release of CK-MB and CK-MB mass (Table 5).

**Table 5:** The relationship between apoptotic index (TUNEL), histopathological myocardial injury score and net release of cardiac marker values in the post-ACC period

**	*CK-MB (U/l)*	*CK-MB mass (µg/l)*	*cTnI (µg/l)*	*hs-cTnT (ng/l)*
Apoptotic index	r = 0.222	r = 0.013	r = 0.283	r = 0.507
(TUNEL)	p = 0.185	p = 0.937	p = 0.090	p = 0.001*
Myocardial	r = 0.260	r = –0.107	r = 0.333	r = 0.416
injury score	p = 0.120	p = 0.530	p = 0.044*	p = 0.010*

ACC: aortic cross-clamping; CK-MB: creatine kinase isoenzyme MB; cTnI: cardiac troponin I; hs-cTnT: high-sensitivity cardiac troponin T; TUNEL: terminal deoxynucleotidyl transferase-mediated deoxyuridine triphosphate nick end-labelling; post-ACC: within 15 minutes of aortic declamping. Net release of cardiac markers was quantified as arteriovenous difference (coronary sinus concentration minus arterial concentration). Relationships between data were tested using Spearman’s correlation analysis. *A *p*-value < 0.05 was considered statistically significant.

We analysed the relationship between apoptotic index, myocardial injury score and intra-operative data. We found a significant positive correlation between apoptotic index and ACC time, CPB time and number of grafted vessels (Table 6). A significant positive correlation was found between myocardial injury score and ACC time, CPB time and number of grafted vessels (Table 6).

**Table 6:** The relationship between apoptotic index (TUNEL), histopathological myocardial injury score and ACC time, CPB time and graft number

	*ACC time (min)*	*CBP time (min)*	*Number of grafted vessel*
Apoptotic index	r = 0.876	r = 0.694	r = 0.445
(TUNEL)	p < 0.001*	p < 0.001*	p = 0.007*
Myocardial	r = 0.867	r = 0.725	r = 0.555
injury score	p < 0.001*	p < 0.001*	p < 0.001*

ACC: aortic cross-clamping; CPB: cardiopulmonary bypass; TUNEL: terminal deoxynucleotidyl transferase-mediated deoxyuridine triphosphate nick end-labelling. Relationships between data were tested using Spearman’s correlation analysis. *A p-value < 0.05 was considered statistically significant.

Additionally, when the relationship between net release of cardiac markers in the post-ACC period and the intra-operative data were analysed, a positive correlation was observed between net release of hs-cTnT and ACC time. Furthermore, a positive correlation was found between net release of hs-cTnT and CPB time (Table 7). No correlation was found between peri-operative data and net release of other cardiac markers in the post-ACC period (Table 7).

**Table 7:** Patient characteristics and demographics

Net release of cardiac markers	ACC time (min)	CPB time (min)	Number of grafted vessel
CK-MB (U/l)	r = 0.110	r = 0.187	r = 0.128
	p = 0.529	p = 0.280	p = 0.448
CK-MB mass (µg/l)	r = 0.110	r = 0.155	r = –0.015
	p = 0.526	p = 0.374	p = 0.931
cTnI (µg/l)	r = 0.157	r = 0.121	r = 0.052
	p = 0.366	p = 0.489	p = 0.759
hs-cTnT (ng/l)	r = 0.448	r = 0.342	r = 0.200
	p = 0.007*	p = 0.047*	p = 0.249

ACC: aortic cross-clamping; CPB: cardiopulmonary bypass; CK-MB: creatine kinase isoenzyme MB; cTnI: cardiac troponin I; hs-cTnT: high-sensitivity cardiac troponin T; post-ACC: within 15 minutes of aortic declamping. Net release of cardiac markers was quantified as arteriovenous difference (coronary sinus concentration minus arterial concentration). Relationships between data were tested using Spearman’s correlation analysis. *A p-value < 0.05 was considered statistically significant.

## Discussion

CABG is a highly complex and risky surgical procedure, and despite well-established myocardial protective procedures, CABG surgery may still cause myocardial damage.^14^ The incidence of PMI varies considerably, from three to 30%, because of different diagnostic criteria and variable patient populations.^15^ Although changes in blood concentrations of cardiac markers, such as CK-MB, myoglobin (Mb) and cardiac troponins are used in the diagnosis of PMI, there are no widely accepted standardised diagnostic criteria.^16^

Myocardial damage causes disruption of the normal cardiac myocyte membrane integrity and loss of intracellular content into the extracellular space. Therefore, elevated levels of cytosolic and structural proteins, such as CK-MB and cardiac troponins, can be detected in the blood.^17^

Interpretation of cardiac biomarkers is difficult after CABG surgery because the specificity of some cardiac markers during CABG surgery is limited, depending on skeletal muscle injury occurring in the surgical procedure. Skeletal muscle injury may increase intra-operative concentrations or activities of some cardiac markers, such as CK-MB and CK-MB mass. As a result, increases in these markers due to skeletal muscle damage may confound the diagnosis of PMI. Consequently, it is important to detect PMI using a highly specific marker.^18^ In this study, the relationship between myocardial apoptosis and injury and the release of biochemical cardiac markers during CABG surgery accompanied by CPB were investigated using histopathological examinations and biochemical measurements.

Although optimal current myocardial protective techniques were applied during surgery in all study populations, our histopathological results revealed that CABG surgery accompanied by CPB and ACC led to slight-to-moderate PMI as well as moderate-to-severe myocardial apoptosis in all cases. Apoptosis is considered one of the mechanisms of cardiomyocyte loss during CPB and cardioplegic arrest during CABG surgery.^19^,^20^ Myocardial apoptosis is induced immediately after cardioplegic arrest and CPB.^21^

Apoptosis during CPB and cardioplegic arrest can be induced by several mechanisms, including I/R injury and the release of cytokines and inflammatory factors.^20^,^22^ Previous studies have reported that cardioplegic cardiac arrest could stimulate pro-inflammatory cytokines, induce cardiomyocytic apoptosis, and impair postoperative cardiac performance.^23^, ^24^ In our study, a positive correlation was found between myocardial apoptotic index and ACC or CPB time. Schmitt *et al.* and Ruifrok *et al.* found a positive correlation between the apoptotic index and duration of ACC and CPB, which was consistent with our study.^25^, ^26^ Conversely, Wu *et al.* reported that myocardial apoptosis showed no correlation with ACC and CPB time.^27^

We observed significant increases in CK-MB, CK-MB mass, cTnI and hscTnT levels within 15 minutes of the reperfusion period. The concentrations of coronary sinus CK-MB, CK-MB mass, cTnI and hscTnT were higher than the corresponding arterial concentrations after aortic declamping, indicating considerable myocardial release of these markers after reperfusion. In addition, we found significant myocardial net release of CK-MB, CK-MB mass, cTnI and hs-cTnT into the coronary circulation after aortic declamping within 15 minutes of reperfusion. Myocardial net release of CK-MB, CK-MB mass, cTnI and hscTnT indicated that myocardial damage had occurred during ACC and cardioplegic cardiac arrest, as well as a very rapid release from the myocardium with the onset of reperfusion.

Although cardiac troponins are structurally bound proteins of striated muscles, the cytosolic pool of cTnT and cTnI may account for the rapid early myocardial release in parallel with CK-MB and CK-MB mass.^28^ Coronary sinus sampling allows direct sampling of the blood draining the heart, therefore the arteriovenous difference provides the closest correlation between cardiac marker release and ischaemic time.

These data obtained during CABG are in accordance with the results of previous studies.^29^,^30^ Bleier *et al.* found significant myocardial net release of CK-MB mass, cTnT and cTnI into the coronary circulation after aortic declamping within 20 minutes of reperfusion.^29^ Koh *et al.* reported that cTnT concentrations increased in every patient after aortic declamping, and were higher in coronary sinus blood than in arterial blood, indicating net myocardial release of troponin T during the period of reperfusion.^30^

Despite the fact that a positive correlation was found between myocardial apoptotic index and CK-MB mass, cTnI and hs-cTnT concentrations, as well as a positive correlation between degree of myocardial injury and cTnI and hs-cTnT concentrations after aortic declamping, the strongest correlation was observed between hs-cTnT and myocardial apoptosis and injury. In a previous study, myocardial biochemical markers demonstrated no correlation with myocardial apoptosis, unlike in our study.^27^

When we examined the relationship between myocardial apoptosis and net release of cardiac markers, a significant positive correlation was found between apoptosis and net release of hs-cTnT after reperfusion. A positive correlation was observed between degree of myocardial injury and net release of cTnI and hs-cTnT, but hs-cTnT demonstrated a stronger correlation with myocardial injury than cTnI after aortic declamping.

We also examined the relationship between ACC and CPB time and net release of cardiac markers. Duration of ischaemic time, which is expected to be a strong predictor of release of biochemical markers, correlated with net release of hs-cTnT but not with that of CK-MB, CK-MB mass and cTnI. In several previous studies, net cTnT release and peak cTnT concentrations were correlated with ACC time, which was consistent with our study.^30^,^31^

Cardiac troponins are highly specific markers of cardiac myocyte damage and seem to better indicate myocardial injury occurring during CABG surgery.^32^-^34^ High-sensitivity troponin assays have recently been developed, being more sensitive than contemporary assays at detecting lower troponin levels. The high-sensitivity troponin T assay has lowered the detection threshold for myocardial necrosis and therefore permits more rapid diagnosis of MI.^35^,^36^

Recently, several studies have been reported related to the ability of hs-cTnT to diagnose PMI after CABG or non-cardiac surgery.^37^,^38^ Wang et al. reported that post-operative hs-cTnT as a parameter alone, independently predicted medium-term mortality and morbidity.^37^ Nagele et al. reported that pre-operative hs-cTnT concentrations were significantly associated with postoperative myocardial infarction and long-term mortality in high-risk patients undergoing major non-cardiac surgery, and they suggested that pre-operatively measured hs-cTnT concentrations may be useful to identify patients at high risk for peri-operative acute MI and increased long-term mortality after non-cardiac surgery.^38^

In our study, although cTnI and hs-cTnT concentrations were well correlated with severity of myocardial injury and apoptosis, hs-cTnT showed a better correlation than cTnI. In addition, hs-cTnT concentrations were correlated with ACC and CPB time. The results of this study indicate that increases in hs-cTnT concentration seem to best reflect myocardial cell damage and apoptosis. Although both cTnI and hs-cTnT may be specific and efficacious markers of myocardial apoptosis and injury occurring during CABG with CPB, hs-cTnT may be a more useful marker than cTnI to detect peri-operative myocardial apoptosis and injury.

## Conclusion

Despite optimal current myocardial protection techniques, PMI may occur during CABG surgery with CPB. Moreover, CABG surgery may cause myocardial apoptosis. Highsensitivity troponin T assay has lowered the detection threshold for myocardial damage and therefore it may provide rapid and specific detection of myocardial injury during CABG surgery with CPB. Measurement of the change in hs-cTnT concentrations may be useful to quantify the severity of perioperative myocardial injury.
